# Using Tomato Recombinant Lines to Improve Plant Tolerance to Stress Combination Through a More Efficient Nitrogen Metabolism

**DOI:** 10.3389/fpls.2019.01702

**Published:** 2020-01-17

**Authors:** María Lopez-Delacalle, Daymi M. Camejo, María García-Martí, Pedro A. Nortes, Manuel Nieves-Cordones, Vicente Martínez, Francisco Rubio, Ron Mittler, Rosa M. Rivero

**Affiliations:** ^1^ Department of Plant Nutrition, CEBAS-CSIC, Murcia, Spain; ^2^ Department of Irrigation, CEBAS-CSIC, Murcia, Spain; ^3^ The Division of Plant Sciences, College of Agriculture, Food and Natural Resources, Christopher S. Bond Life Sciences Center, University of Missouri, Columbia, MO, United States; ^4^ University of Missouri School of Medicine, Christopher S. Bond Life Sciences Center, University of Missouri, Columbia, MO, United States

**Keywords:** nitrogen metabolism, abiotic stress combination, tomato recombinant lines, photosynthesis, chlorophyll fluorescence, ionomics, amino acids, gene expression

## Abstract

The development of plant varieties with a better nitrogen use efficiency (NUE) is a means for modern agriculture to decrease environmental pollution due to an excess of nitrate and to maintain a sufficient net income. However, the optimum environmental conditions for agriculture will tend to be more adverse in the coming years, with increases in temperatures, water scarcity, and salinity being the most important productivity constrains for plants. NUE is inherently a complex trait, as each step, including N uptake, translocation, assimilation, and remobilization, is governed by multiple interacting genetic and environmental factors. In this study, two recombinant inbred lines (RIL-66 and RIL-76) from a cross between *Solanum lycopersicum* and *Solanum pimpinellifoilum* with different degree of tolerance to the combination of salinity and heat were subjected to a physiological, ionomic, amino acid profile, and gene expression study to better understand how nitrogen metabolism is affected in tolerant plants as compared to sensitive ones. The ionomics results showed a different profile between the two RILs, with K^+^ and Mg^2+^ being significantly lower in RIL-66 (low tolerant) as compared to RIL-76 (high tolerant) under salinity and heat combination. No differences were shown between the two RILs in N total content; however, N-NO_3_^−^ was significantly higher in RIL-66, whereas N-N_org_ was lower as compared to the other genotype, which could be correlated with its tolerance to the combination of salinity and heat. Total proteins and total amino acid concentration were significantly higher in RIL-76 as compared to the sensitive recombinant line under these conditions. Glutamate, but more importantly glutamine, was also highly synthesized and accumulated in RIL-76 under the combination of salinity and heat, which was in agreement with the upregulation of the nitrogen metabolism related transcripts studied (*SlNR*, *SlNiR*, *SlGDH*, *SlGLT1*, *SlNRT1.2*, *SlAMT1*, and *SlAMT2*). This study emphasized the importance of studying abiotic stress in combination and how recombinant material with different degrees of tolerance can be highly important for the improvement of nitrogen use efficiency in horticultural plants through the targeting of N-related markers.

## Introduction

Environmental conditions will tend to be more adverse in the coming years due to global warming, with increases in temperatures, water scarcity, and salinity. The climate predictions are devastating for agriculture, which point to plant growth inhibition and a reduction in productivity to never-before-seen maximum levels. Moreover, it has been shown that when these devastating conditions act in combination, i.e., salinity plus high temperature, the economic losses in agriculture triple (Rizhsky et al., 2004; Mittler, 2006; Suzuki et al., 2014). This situation makes necessary to act urgently in order to ensure that there will be enough food for the growing world population. Thus, the study of the physiological, biochemical, and molecular mechanisms implicated in the plant's response to abiotic stress are crucial. In the last few years, the study of the plant's response to abiotic stresses acting in combination has become important in the elucidation of the mechanisms implicated in the plant's tolerance and their interconnections within plant metabolism (Rivero et al., 2014; Martinez et al., 2016; Zandalinas et al., 2016; Martinez et al., 2018). Martinez et al. (2016) demonstrated that when tomato plants were subjected to the combination of salinity and heat, the metabolic, biochemical, and molecular profile obtained was different from that observed when these stresses were applied individually. Similarly, Rivero et al. (2014) concluded that there was a specific accumulation of osmoprotectants, more specifically, trehalose and glycine betaine, when tomato plants were grown under the combination of salinity and heat, that was not observed when applying the single stresses.

Aside from the different responses observed under stress combination as compared to single stresses, it was found that when abiotic stresses acted jointly, antagonistic responses could also be observed (Cheong et al., 2002; Rizhsky et al., 2004; Mittler, 2006). For example, when plants were subjected to heat stress, their stomata were opened to reduce leaf temperature. But, if at the same time these plants were subject to drought, their stomata won't be able to be opened, and an increase in the leaf temperature and cell death were observed.

Nitrogen (N), together with phosphorus and potassium, is one of the primary macronutrients needed by plants for their survival. During the last 50 years, the application of N fertilizers has increased 20 times and its application is expected to increase to 180 million tons by 2030 (Spiertz, 2009; Rütting et al., 2018). Also, the prices of N fertilizers have risen more than 2.5 times in the last decade (Spiertz, 2009). This has caused problems to the farmers' costs and also to the environment. Thus, the development of plant varieties with a better nitrogen use efficiency (NUE) may be a mean for modern agriculture to decrease environmental pollution due to the excess use of nitrates and to maintain a sufficient N net income. NUE is inherently a complex trait, as each step, including N uptake, translocation, assimilation, and remobilization, is governed by multiple interacting genetic and environmental factors.

N enters to the root through an active symport mechanism that is dependent on nitrate or ammonium transporters (NRTs or AMTs, respectively) with H^+^ and ATP consumption by the activity of an ATPase H^+^-P. This NO_3_^−^ can be stored in the vacuole or reduced to NO_2_^−^ by the enzyme nitrate reductase (NR) and subsequently to NH_4_^+^ by the enzyme nitrite reductase (NiR). This NH_4_^+^ is then added to C skeletons within the N assimilation pathway, resulting in the synthesis of certain important amino acids for plant metabolism. The remaining NO_3_^−^ is transported by the xylem to mesophyll cells, where it is temporarily stored in the vacuole (Masclaux-Daubresse et al., 2010). The NH_4_^+^ ions resulting from NO_3_^−^ reduction (or those directly incorporated into the cells from the soil environment) serve as a substrate for the enzymes glutamine synthetase (GS) and glutamate synthase (GOGAT) to produce glutamic acid (Glu) and glutamine (Gln), both of which are precursors for the synthesis of the rest of the amino acids (Suzuki and Knaff, 2005).

It is known that N metabolism and its remobilization processes within the plant are regulated by internal factors, such as NR and GS activities (Tian et al., 2016) and by external factors, such as the abiotic stresses of heat, drought, and salinity (Zandalinas et al., 2018). Hossain et al. (2012) demonstrated that tomato plants subjected to salinity conditions, in addition to limiting NO_3_^−^ acquisition by the roots, also restricted their ability to reduce and assimilate N by inhibiting the synthesis and activities of the assimilation enzymes, such as NR, NiR, GS, and GOGAT. NR activity under saline stress conditions decreases significantly in tomato (Debouba et al., 2007), sugar beet (Ghoulam et al., 2002) maize (Baki et al., 2000), and beans (Gouia et al., 1994). However, it has been shown that salinity can also have a stimulating effect on NR activity in the roots of tomato (Debouba et al., 2006) or soybean (Bourgeais-Chaillou et al., 1992). Similarly, heat stress decreases the levels of absorption of nutrients and assimilation proteins in tomato plants (Giri et al., 2017), limiting the development, growth, and reproduction of plants (Boyer, 1982; Zinn et al., 2010; Williams et al., 2013). Heat stress can also alter the enzymes involved in N metabolism, altering the assimilation of NO_3_^−^ and NH_4_^+^(Klimenko et al., 2006; Hungria and Kaschuk, 2014; Giri et al., 2017). In this sense, Argentel-Martínez et al. (2019) concluded that heat stress in wheat alters the activity of the enzymes NR and GS involved in the assimilation of N. When abiotic stresses act in combination, these results could change, and could be very different that the observed under the single stress described previously. For example, Jin et al. (2016) found that in *Portulaca oleracea* L. plants there was a specific accumulation of certain amino acids, such as Gln, ornithine, tyrosine (Tyr), valine, and tryptophan (Trp), when subjected to drought and heat stress acting in combination, which suggests that the plant is making a cellular osmotic adjustment aimed at maintaining leaf turgor during this particular stress condition. It has also been shown that under drought or UV-B stress, cucumber plants increased the activity of the NR enzyme, while decreasing their total cellular NO_3_^−^ content (Rybus-Zając and Kubiś, 2018).

At present, there are very little information about the effect of abiotic stress combination on the N uptake and assimilation pathway (Cui et al., 2019). Thus, in order to identify agronomic strategies that can be used to fight against the consequences of climate change in crops, detailed studies related to nitrogen metabolism should be developed at the physiological, biochemical, and molecular levels, which is also the primary metabolism related to growth and production in plants. The results from these studies will provide data on the agronomic responses to the environmental scenario predicted for the coming years. On the other hand, the use of varieties with different sensitivity to the abiotic stresses acting jointly could be a great strategy to identify these tolerance-related mechanisms. Traditionally, the selection of the different agronomical plant varieties has been done accordingly to their vigor and yield. However, the new climate scenario is forcing us to select plant varieties based on their tolerance to new environmental conditions. Thus, in this study, two recombinant lines (RIL-66 and RIL-76) from a cross between *Solanum lycopersicum* and *Solanum pimpinellifoilum* with different degrees of tolerance to the combination of salinity and heat were subjected to a physiological, ionomic, amino acids profile and gene expression study related to the N acquisition and assimilation pathway to better understand how nitrogen metabolism may be affected by stress combination. The identification of some metabolic and/or molecular markers may help to advance knowledge on how to improve NUE in plants for an improved and more sustainable agriculture.

## Materials and Methods

### Plant Material and Growth Conditions for the Preliminary Recombinant Inbred Lines Selection Experiments

A scheme summarizing the experimental design followed for the selection of sensitive and tolerant tomato RILs to the combination of salinity and heat can be found in Supplementary Material (**Supplemental Scheme 1**). In brief, our experiments began with a population of 84 recombinant inbred lines (RILs) resulting from a cross between *S. lycopersicum* (CLN2498E) x *Solanum pimpinellifolium* (LA1579). This recombinant inbred collection was kindly ceded by Prof. Perez-Alfocea (CEBAS-CSIC, Spain). All seeds were germinated in vermiculite and irrigated with distilled water until 95% of the seeds had germinated. Seven days after germination all seedlings were transferred to two different greenhouse modules (module A and B). Nine plants from each recombinant line were transplanted into rock wool sacs (1 m long; three plants per sac). Plants were irrigated with a modified Hoagland's solution with the following composition: KNO_3_ (3 mM), Ca(NO_3_)_2_ (2 mM), MgSO_4_ (0.5 mM), KH_2_PO_4_ (0.5 mM), Fe-EDTA (10 µM), H_3_BO_3_ (10 µM), MnSO_4_·H_2_O (1 µM), ZnSO_4_·7H_2_O (2 µM), CuSO_4_·5H_2_O (0.5 µM), and (NH_4_)_6_Mo_7_O_24_·4H_2_O. The pH and electric conductivity (EC) of the nutrient solution were measured and maintained within the range of 5.2–5.6 and 1.4–1.7 mS/cm, respectively. The environmental conditions of each greenhouse module were the same for 10 days (25°C/20°C day/night, 65–75% RH). The greenhouse climate control system operated as described in Rodriguez-Ortega et al. (2017). After 10 days under these conditions, the treatments were started. For these, the air temperature of module B was increased to 35°C/20°C (day/night) and half of the plants growing in this received 75 mM NaCl in their irrigation solution. Therefore, the plants growing in module A were considered as the control plants, and plants growing in module B were considered as the stress combination (salinity+heat) treated plants. The treatments were maintained for 2 weeks, after which the plants were sampled. The fresh weight (FW) and the dry weight (DW) of the shoots were determined. For the DW, the plant material was oven-dried for 72 h at 70°C and weighed. With the values obtained for FW % tolerance was determined as follows:

%tolerance=100−(FW control-FW stress)/FWcontrol

**Figure 1:**
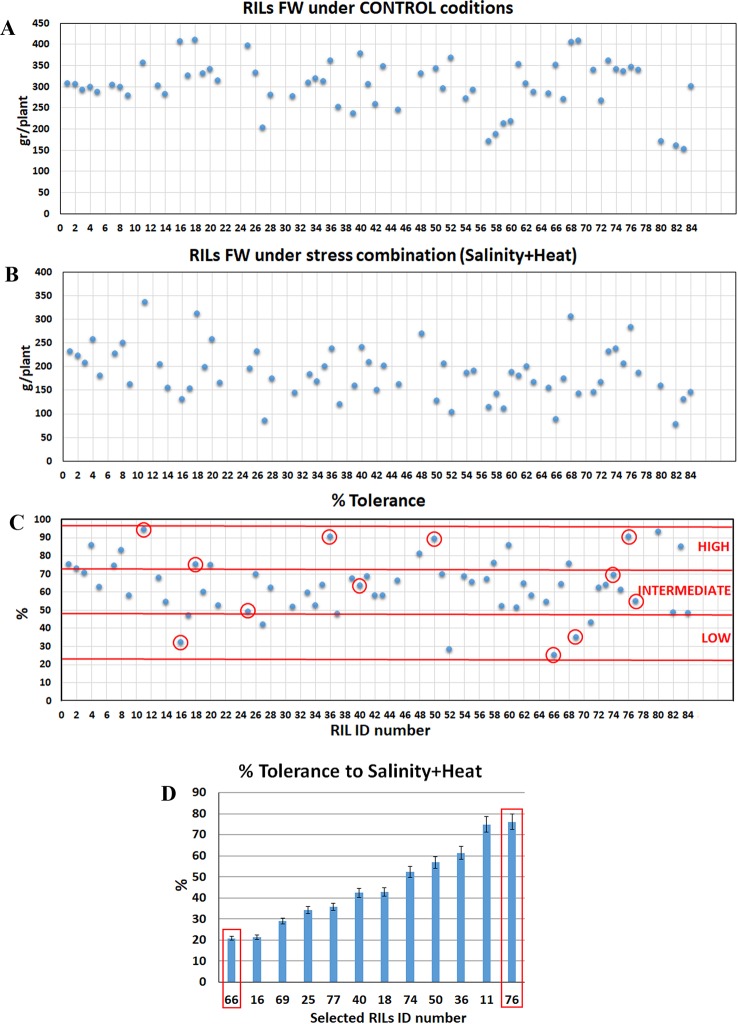
Tomato recombinant inbred lines (RILs) selection for their tolerance to the combination of salinity and heat. Eighty four RILs lines were grown under control **(A)** and under the combination of salinity and heat **(B)** and fresh weight (FW) was recorded. Twelve RILs were selected for each grade of tolerance (low, intermediate, and high tolerance) to the combination of salinity and heat as compared to control plants **(C)**. The selected lines (C, red circle) were grown again under growth chamber conditions to validate the different grade of tolerance obtained previously under greenhouse conditions. RIL-66 (low tolerant) and RIL-76 (high tolerant) were selected for further experiments **(D)**. Values represented in each figure are means ± SE (n = 6).

From the results obtained (**Figures 1B, C**), three levels of tolerance were established: low, intermediate, and high tolerance to the combination of salinity+heat. Then, 12 RILs (4 lines from each level of tolerance) were selected and their percentage of tolerance to the stress combination was validated under growth chamber conditions. Plants were germinated as described previously. Seven days after germination all seedlings were transferred to growth chamber (chamber A) with 16/8 h photoperiod (day/night) at 25°C/20°C (day/night) and 65% RH. Plants were grown in 20 L containers filled with modified Hoagland nutrient solution. The composition, chemical concentration, pH, and CE used for the nutrient solution for plant irrigation were as described previously. After 8 days of the transplanting and 15 days after germination, salinity and temperature treatments were imposed. For this, half of the plants from each RILs were transferred to another growth chamber (chamber B) where the temperature was set at 35°C/20°C (day/night) and the irrigation nutrient solution was supplemented with 75 mM NaCl. After 15 days of growing under these conditions, plants were sampled and % tolerance to salinity+heat was calculated as described previously. After validating the results obtained for % tolerance, two RILs were selected for the experiments: the ones having the lowest (RIL-66) and the highest (RIL-76) % tolerance to the combination of salinity+heat.

### Experimental Design and Plant Sampling

Two recombinant lines (RIL-66 and RIL-76) differing in their tolerance to the combination of salinity and heat were selected for this experiment as described previously. Germination and growth chamber conditions were the same as described previously for chamber A for 8 days. After 8 days of transplanting and 15 days after germination, salinity and temperature treatments were imposed. Half of the plants were transferred to another growth chamber (chamber B) with the same environmental conditions as those described previously but with an air temperature set at 35°C/20°C (day/night). In parallel, half of the plants from each growth chamber received 75 mM NaCl in their nutrient solution, so this time individual stresses were also applied in order to better dissect the specificity of the stress combination. Thus, the treatments that were applied were: a) control (25°C 0 mM NaCl), b) salinity (25°C and 75 mM NaCl), c) heat (35°C 0 mM NaCl), and d) heat+salinity (35°C and 75 mM NaCl).

Plants were grown under these conditions for 10 days, and at the end of the experiments, leaves, and roots were sampled. For each determination described below six biological replicates for each treatment were used.

### Gas Exchange and Chlorophyll Fluorescence Parameters

The different photosynthetic and chlorophyll fluorescence parameters were measured at the end of the experiment in the youngest fully-expanded leaf of each plant (six plants per treatment), using a LI-6400XT photosynthesis system (Li-Cor, Inc., Lincoln, NE, USA). The conditions established in the LI-COR were as described in Martinez et al. (2018).

### Mineral Nutrients and Anions Analysis

0.1 g of dried plant material from six biological replicates were digested with HNO_3_:HClO_4_ (2:1, v:v), and cations concentrations were determined by atomic absorption spectrometry with an ICP-OES (Iris Intrepid II, Thermo Electron Corporation, Franklin, USA). Cations concentration were expressed as log_2_ of the normalized values against control plants. Absolute values obtained for the concentration of the main cations can be found in **Supplementary Table S1**.

### Hydrogen-Nuclear Magnetic Resource Analysis for Amino Acid Concentration

Six biological replicates of tomato leaves for each treatment were used for metabolite profiling by ^1^H-NMR. The extraction and quantification of the amino acid were performed as described in Blanco-Ulate et al. (2015). Amino acids concentration was expressed as log_2_ of the normalized values against control plants. Absolute values obtained for the concentration of the amino acids assayed can be found in **Supplementary Table S2**.

### Enzymatic Activities

#### Crude Extract

For the determination of the activities of NR, NiR, glutamate dehydrogenase (GDH), and GOGAT, leaf tissues from six biological replicates were homogenized with 50 mM KH_2_PO_4_ buffer (pH 7.5), containing 2 mM ethylenediaminetetraacetic acid (EDTA), 2 mM DL-dithiothreitol (DTT), and 1% polyvinylpolypyrrolidone (PVPP). For the determination of the activity of GS, leaf tissues were homogenized with 50 mM Tris-HCl (pH 7.5) containing 5 mM EDTA, 1 mM DTT, and 1% of PVPP. The homogenate was centrifuged at 15,000 rpm for 20 min and the supernatant collected was used for measuring the enzyme activity. Total soluble proteins were measured with the Bradford method (Bradford, 1976).

#### Nitrate Reductase Assay

NR (EC 1.7.1.1) activity was measured by the decrease in absorbance (A_340_) due to NADH oxidation (Campbell and Smarrelli, 1978).

#### Nitrite Reductase Assay

NiR activity was determined by the absorbance change at 540 nm as described by Lillo (1984).

#### Glutamate Synthase/Glutamine Synthase Assays

GOGAT (EC 2.6.1.53) activity was assayed by determining the rate of glutamine-dependent NADH oxidation as described by Groat and Vance (1981) with some modifications. The reaction assay was carried out in a mixture reaction containing 25 mM KH_2_PO_4_ buffer (pH 7.5), 2.5 mM 2-oxoglutarate, 1 mM hydroxylamine, 0.1 mM NADH, and 50–100 µg total protein, with the reaction started by the addition of 10 mM of L-glutamine. GOGAT activity was assayed by determining the rate of glutamine-dependent NADH oxidation at 340 nm for 180 s.

GS (EC 6.3.1.2) activity was assayed according to Slawyk and Rodier (1986) with some modification. Enzymatic extract (150 µg of total protein) was incubated in a mixture reaction containing 15 mM magnesium chloride, 0.5 mM EDTA, 20 mM potassium-glutamate, 4 mM ammonic acetate, and 1 mM adenosine triphosphate (ATP) at 30°C for 15 min. The reaction was stopped by the addition of 1 N sulfuric acid and then the product was centrifuged at 11,000 rpm for 2 min. GS activity was determined by measuring the ATP-dependent phosphate concentration. The phosphate concentration was measured with a Phosphate Assay kit (Sigma-Aldrich) at 620 nm. A phosphate standard curve was used to determine the amount of free phosphate in the sample.

#### Glutamate Dehydrogenase Assay

GDH (EC 1.4.1.2) activity was assayed by measuring the rate of 2-oxoglutarate-dependent NADH oxidation at 340 nm for 180 s (Robinson et al., 1991)

### Gene Expression

Total RNA was isolated from whole tomato leaves from six biological replicates, as described by Martinez et al. (2018). The primer sequences used for the different genes tested as well as the primer sequences of the internal controls are available in **Supplementary Table S3**. As reference gene, SlEF1α was used as described previously by Lacerda et al. (2015) and Cheng et al. (2017). The normalization of transcript expression against housekeeping genes, reaction components, and PCR settings were carried out as described by Martinez et al. (2018). Expression and log_2_ values of the different genes can be found in **Supplementary Table S4**.

### Statistical Analysis

The software package SPSS^®^ (ver.10.0, SAS Institute) was used for statistical analyses of the above-mentioned parameters. A two-way ANOVA using Tukey's test was used to compare between treatments and recombinant lines for significant differences at *p < 0.05* for all cases and standard error (SE) values for the different treatments and genotypes was calculated and added as shown in figures.

## Results

### Recombinant Inbred Lines Selection Under the Combination of Salinity and Heat

Eighty-four tomato recombinant inbred lines (from here on RILs) were grown in two independent greenhouses at different environmental conditions: A) control conditions (25°C, 16/8 h day/night photoperiod, 65% relative humidity) where the plants were irrigated with a modified Hoagland's nutrient solution and 0 mM NaCl; and B) salinity+heat conditions (35°C, 16/8 day/night photoperiod, 65% relative humidity) where the plants were irrigated with a modified Hoagland's solution supplemented with 75 mM NaCl. Plants were grown under these conditions for 1 month and fresh weight and dry weight of the plants were recorded (**Figure 1**). In order to evaluate the degree of tolerance of each line to the combination of salinity and heat, the percentage of tolerance was calculated as described in the *Materials and Methods* section (**Figure 1C**). After analyzing the results obtained and shown in **Figure 1C**, three different groups were identified: lines with low tolerance (from 25 to 50% tolerance) intermediate tolerance (from 50 to 75% tolerance) and high tolerance (from 75 to 100% tolerance).

From each group, 4 plants were selected (a total of 12 plants) for the next experiment (marked with a red circle, **Figure 1C**) based on the fresh weight obtained under control conditions, which had to be 300 g per plant or higher (fresh weight average obtained under control conditions). These 12 lines were grown again in hydroponics, but this time in two growth chambers, with same characteristics as those mentioned above for the greenhouses (see full description in *Materials and Methods* section) in order to have full control of the environmental and nutrient solution conditions. As **Figure 1D** showed, the results for the % tolerance obtained in the growth chambers for these lines were very similar to those obtained previously in the greenhouse experiment. Based on these results, a RIL line with a low tolerance (RIL-66) and a RIL line with high tolerance (RIL-76) to the combination of salinity and heat, were selected in order to elucidate the mechanisms implicated in this tolerance.

**Figure 2:**
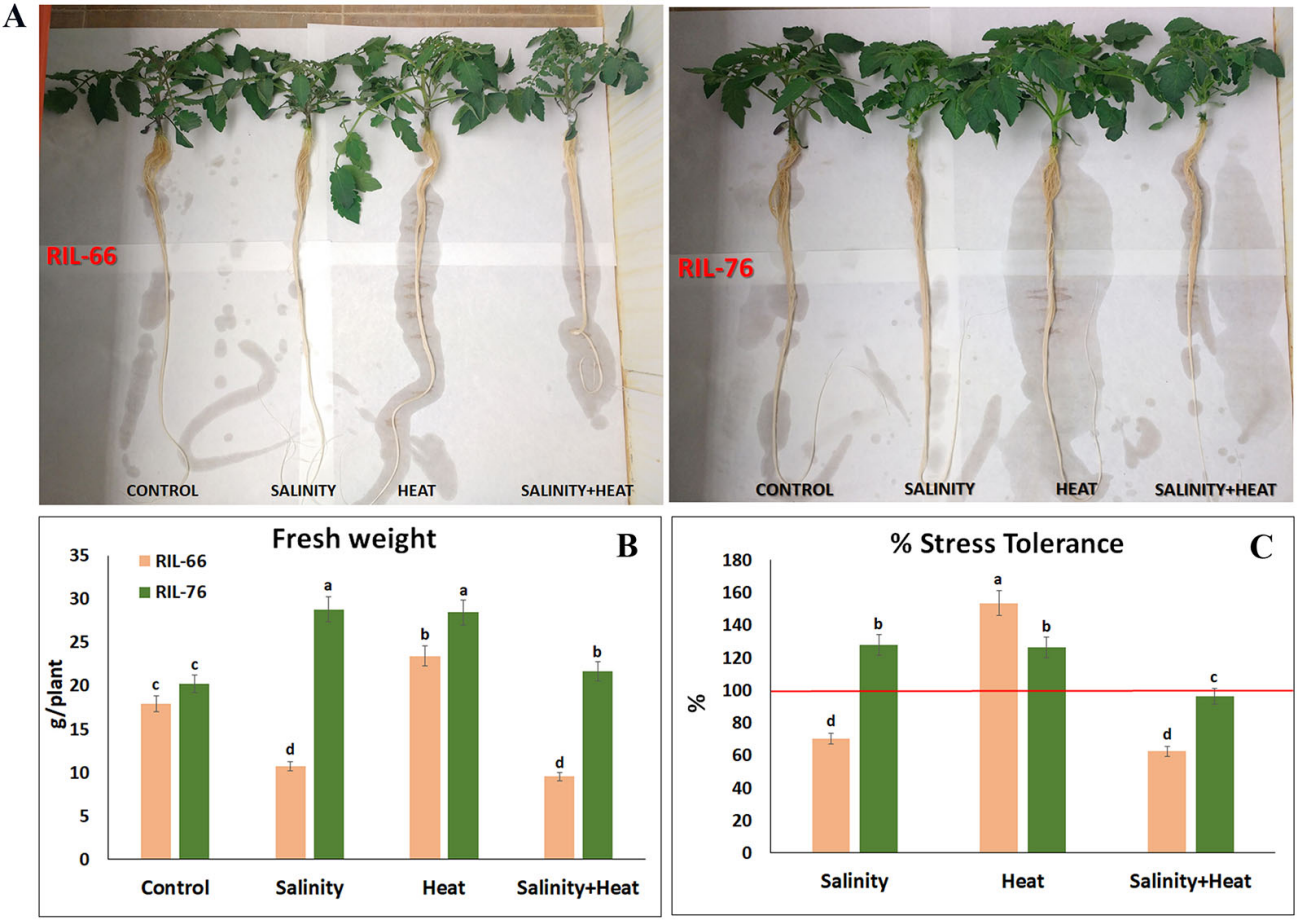
Phenotypes and tolerance rate in tomato recombinant inbred line (RIL)-66 and RIL-76. **(A)** Phenotypes obtained in tomato RIL-66 and RIL-76 under control, salinity, heat stress, and the combination of salinity + heat stress. **(B)** Fresh weight (FW) of the tomato RIL-66 and RIL-76 at the end of the experiment (15 days of treatments). **(C)** Percentage of tolerance to salinity, heat, and the combination of salinity and heat obtained in RIL-66 and RIL-76 respect to their controls (red line = 100% of tolerance). Bars are mean values ± SE (n = 6), and bars with different letters are significantly different at p < 0.05 according to Tukey's test. (n = 6).

In a third experiment, RIL-66 (sensitive to salinity+heat combination) and RIL-76 (high tolerant to salinity+heat combination) were both grown in two different growth chambers set with the same environmental conditions, except for the temperature, set at 25°C in chamber A and 35°C in chamber B. Also, half of the plants in each chamber received 75 mM NaCl in their nutrient solution. Therefore, in this experiment, four treatments were applied: control (25°C + 0 mM NaCl), salinity (25°C + 75 mM NaCl), heat (35°C + 0 mM NaCl), and salinity+heat (35°C + 75 mM NaCl), in order to characterize the specificity of the tomato plants response to the combination to salinity and heat as compared to the single stress responses (**Figure 2**).

As shown in **Figure 2A**, under control conditions, RIL-66 showed a similar phenotype as RIL-76, which was confirmed with the fresh weight data obtained for these plants. Thus, under control conditions, RIL-66 and RIL-76 did not show significant differences in their fresh weight (**Figure 2B**). However, under salinity, heat, and the combination of salt+heat, RIL-76 had a significant biomass increase with respect to RIL-66 (**Figure 2A**), being three times higher (in case of salinity and salt+heat) and two times higher (in case of heat) as compared to the biomass obtained in RIL-66 under these conditions (**Figure 2B**). These data translated in an increase in the percentage of stress tolerance of RIL-76 as compared to RIL-66 (**Figure 2C**). Interestingly, RIL-76 had an increased biomass under salinity and under heat with respect to the same plants grown under control conditions (**Figure 2A**), and similar observations were made for RIL 66 under heat, which translated into a higher percentage of tolerance to these stresses with respect to their controls threshold (100%) (**Figure 1C**). Lastly, it should be highlighted that RIL-76 showed 100% tolerance under the combination of salinity and heat, which meant that this line was able to maintain a good biomass production under stress combination conditions.

### Photosynthetic Performance

**Figure 3:**
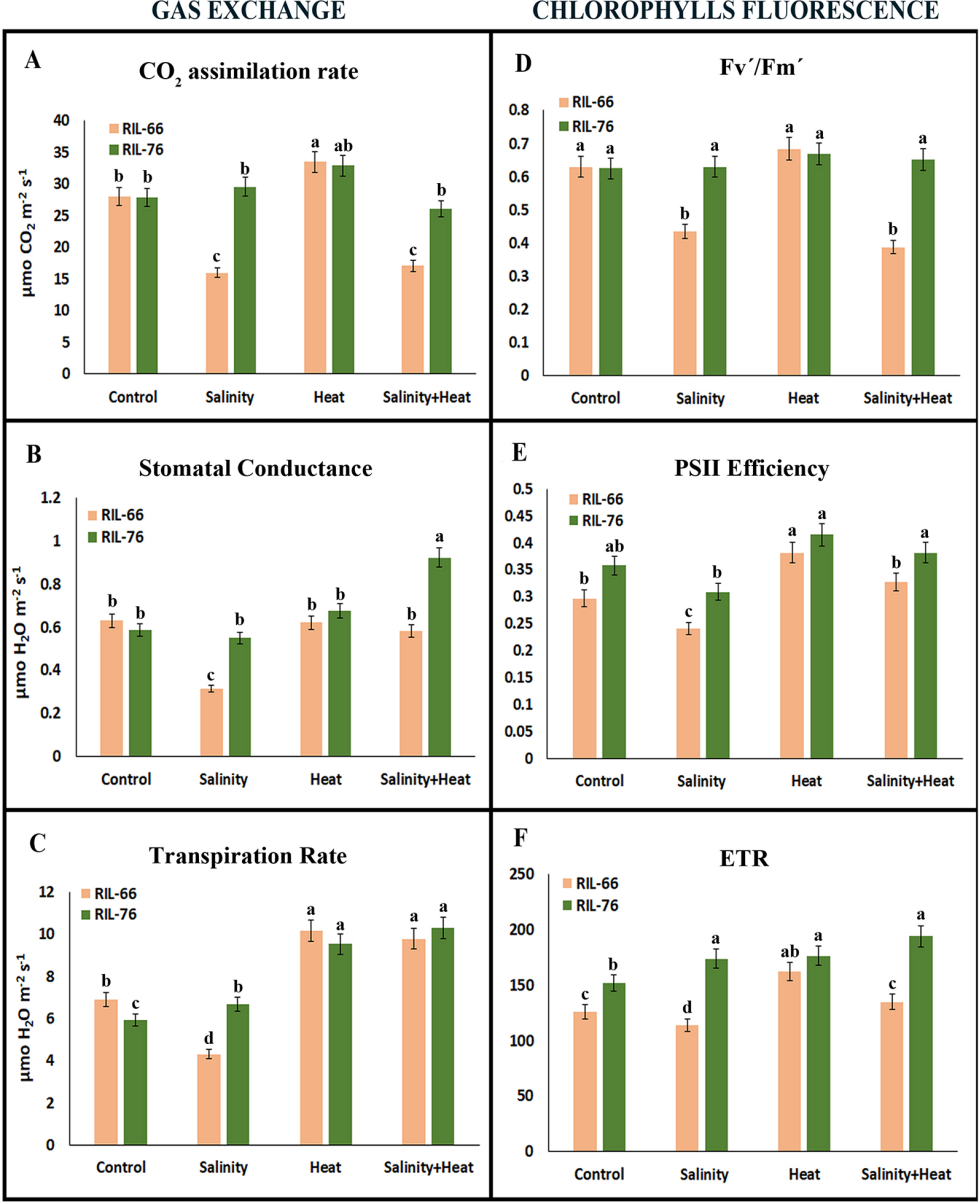
Gas exchange parameters **(A**–**C)** and chlorophyll fluorescence parameters **(D**–**F)** in tomato recombinant inbred line (RIL)-66 and RIL-76 under control, salinity, heat stress, and the combination of salinity and heat. Each figure is representative of three different measurements during the experiment. Bars are mean values ± SE (n = 6), and bars with different letters are significantly different at p < 0.05 according to Tukey's test. (n = 6).

There are several physiological stress markers that can be measured in plants and that can provide insights on how photosynthesis and photosynthetic components behave under stress conditions, such as gas exchange parameters (CO_2_ assimilation rate, stomatal conductance, transpiration rate), and chlorophyll fluorescence parameters (Fv/Fm, ΦPSII, and ETR) (**Figure 3**). In relation to gas exchange parameters (**Figures 3A**–**C**), the CO_2_ assimilation rate was higher in RIL-76 as compared to RIL-66 for salinity and the combination of salinity and heat treatments, obtaining values for RIL-76 that were similar to those obtained in control plants for this parameter under these two treatments (**Figure 3A**). The heat treatment induced an increase in the CO_2_ assimilation rate of about 25% with respect to control plants in both recombinant lines studied. On the contrary, salinity or the combination of salinity and heat reduced net photosynthesis by 42 and 35%, respectively, in RIL-66 as compared to control plants (**Figure 3A**).

Similar results to those observed for net photosynthesis were also obtained for stomatal conductance (**Figure 3B**). Curiously, the transpiration rate was higher and similar in both recombinant lines under heat and the combination of salinity and heat treatments (**Figure 3C**) with respect to control plants. Under control conditions, RIL-76 had a significant lower transpiration rate as compared to RIL-66. However, the lowest transpiration values were obtained when salinity was applied as a sole stress in the sensitive line RIL-66.

Fluorescence of the chlorophylls has been commonly used as a good marker for quantifying damage due to stress in plants. In this sense, quantum yield efficiency (Fv´/Fm´), PSII efficiency (ɸPSII), and electron transport rate (ETR) were measured in our sensitive and tolerant recombinant lines under simple or combined stress conditions (**Figures 3D, E**). Quantum yield efficiency (Fv´/Fm´) showed a similar response as that observed for the net photosynthesis for both lines, sensitive and tolerant (**Figure 3D**), with the most significant differences observed when salinity and salinity+heat were applied as compared to control or heat-stressed plants. PSII efficiency was lower in the sensitive line RIL-66 than in the tolerant RIL-76 line under control condition, which may be indicative of an important physiological difference between these two tomato lines (**Figure 3E**). Salinity was again the stress that resulted in the lowest values of the chlorophyll fluorescence parameters in the sensitive line as compared to the tolerant. Lastly, the ETR showed similar values to those observed for PSII efficiency in both recombinant lines under the different stresses applied (**Figure 3F**).

### Cations and Nitrogen Forms Content

**Figure 4:**
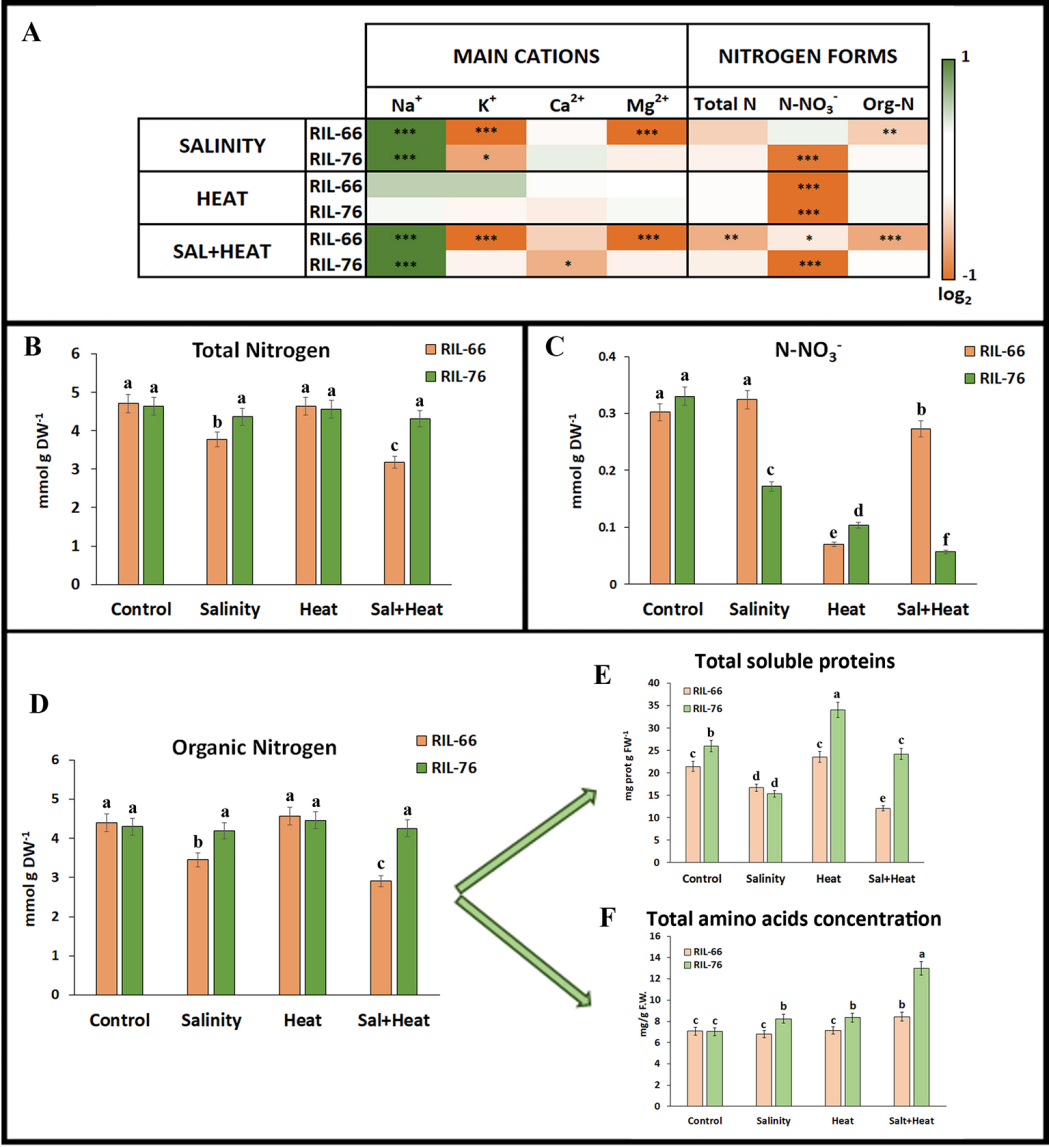
Ionomics in leaves of tomato recombinant inbred line (RIL)-66 and RIL-76 under salinity, heat, or the combination of salinity and heat. **(A)** Heat map of the concentration (expressed as log2) of the main cations and nitrogen (N) forms in tomato leaves of RIL-76 (salinity+heat tolerant) and RIL-66 (salinity+heat sensitive) under salinity, heat stress, and the combination of salinity+heat as compared to their controls. (**B**–**F**) Absolute concentration of total nitrogen **(B)**, nitrates **(C)**, organic nitrogen **(D)**, soluble proteins **(E)**, and total amino acids **(F)** obtained in RIL-66 and RIL-76. Bars are mean values ± SE (n = 6), and bars with different letters are significantly different at p < 0.05 according to Tukey's test. (n = 6). Stars are representative of significant differences p < 0.05 (*p < 0.05, **p < 0.01, ***p < 0.001) respect to control plants.

Salinity, heat, or their combination have also been known to induce ionic and other nutrient imbalances in plants, with NO^−^_3_ being one of the nutrients that is most affected by Na^+^ intracellular concentration. With the aim of observing if there were any differences in the leaf's ion concentration between a sensitive or tolerant tomato plant under the different stresses applied, but especially under the combination of salinity+heat, the most important physiologically-active cations (as Na^+^, K^+^, Ca^2+^, and Mg^2+^) and different nitrogen forms (total N, NO^−^_3_, and organic N) were measured (**Figure 4**). As was expected, Na^+^ concentration was higher in the treatments where NaCl was applied exogenously (salinity and salinity+heat) in both recombinant lines as compared to their controls. As shown in **Figure 4A**, the ionomic profile obtained for each of the two RILs was different depending on the type of stress applied, with K^+^ and Mg^2+^ concentrations being significantly lower in the sensitive RIL-66 line as compared to the tolerant RIL-76 line under salinity or salinity+heat combination (**Figure 4A**; **Supplementary Table S1**). K^+^ concentration was significantly lower in RIL-76 under salinity as compared to control plants, but higher than the concentrations found in RIL-66. On the other hand, Ca^2+^ only showed a significant lower concentration in the tolerant RIL-76 line under the combination of salinity and heat (**Figure 4A**; **Supplementary Table S1**).

With respect to the different nitrogen forms found in these recombinant lines, total N content and organic nitrogen (**Figures 4A, B, D**) showed significant differences between the tolerant and the sensitive recombinant lines only when salinity and heat were applied in combination, with concentrations for these two nitrogen forms significantly lower in the sensitive RIL-66 line. Also, organic N was lower in RIL-66 under salinity as compared to control plants. On the contrary, N-NO_3_^−^ was significantly lower in RIL-76 under any of the stresses studied with respect to control plants, whereas in the sensitive RIL-66 line, the N-NO_3_^−^ concentration was significantly lower than control plants when salinity and heat were applied in combination, but moreover when heat was applied as a single stress (**Figures 4A, C**).

**Figure 5:**
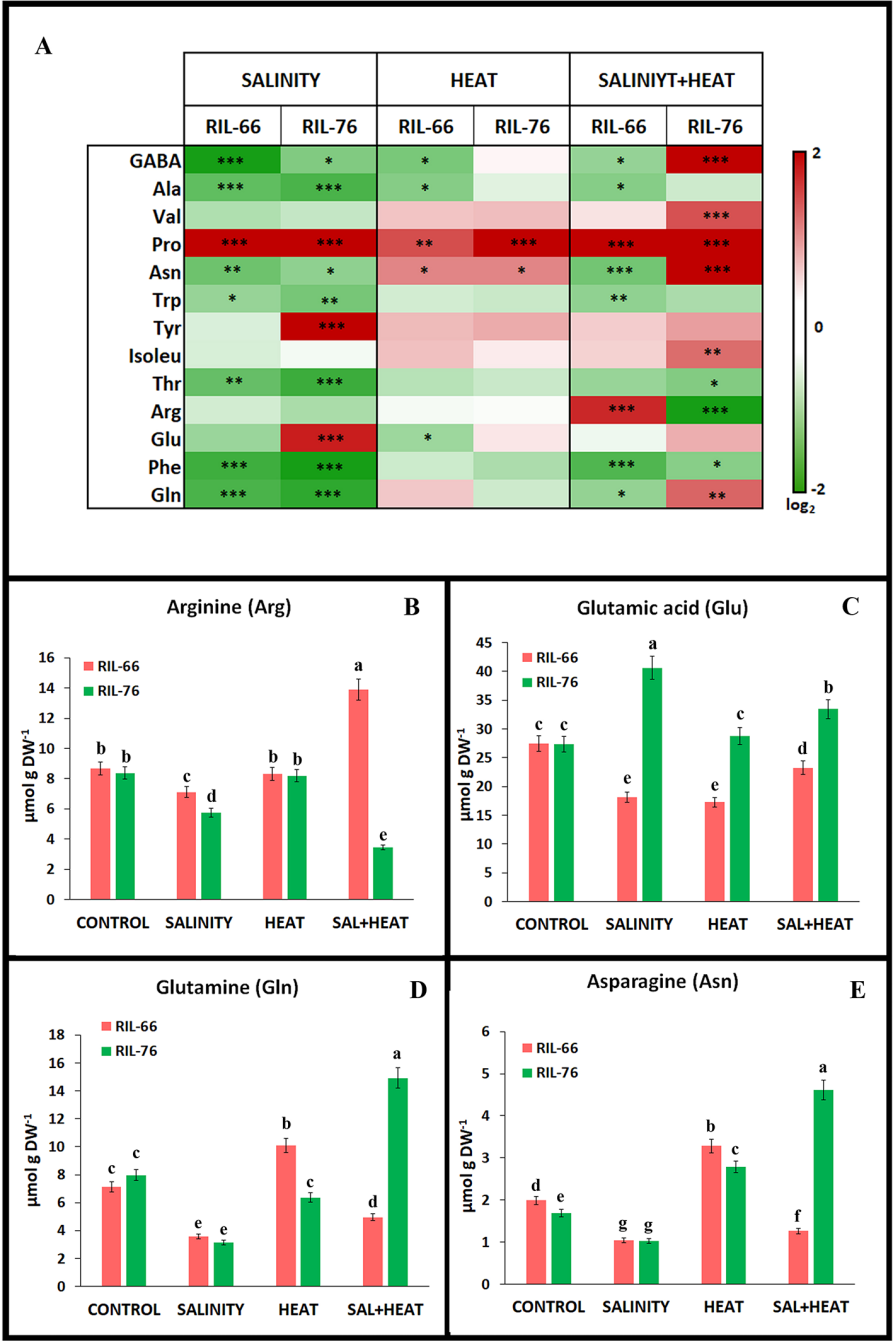
Amino acids concentration in leaves of tomato recombinant inbred line (RIL)-66 and RIL-76 under salinity, heat, or the combination of salinity and heat. **(A)** Heat map of the amino acid concentration (expressed as log_2_) in tomato leaves of RIL-66 (salinity + heat sensitive) and RIL-76 (salinity + heat tolerant) under salinity heat and the combination of salinity+heat. Stars are representative of significant differences p < 0.05 (*p < 0.05, **p < 0.01, ***p < 0.001) respect to control plants. **(B**–**E)** Absolute concentration of the main amino acids related to nitrogen assimilation pathway. Bars are mean values ± SE (n = 6), and bars with different letters are significantly different at p < 0.05 according to Tukey's test. (n = 6).

Proteins and amino acids are the most important and the major sources of organic nitrogen in plants, and for this reason these were an object of our study (**Figures 4E, F**). Total soluble proteins were significantly higher in the tolerant RIL-76 under heat stress as compared to control plants and to the sensitive RIL-66 plants (**Figure 4E**). Salinity induced a reduction in the total soluble proteins in both RIL-66 and RIL-76. However, when salinity and heat were applied together, RIL-66 showed an important reduction of more than 50% with respect to control plants, whereas the total soluble protein concentration found in RIL-76 was similar to that obtained in control plants (**Figure 4E**). In the case of total amino acids concentration, RIL-76 had a higher concentration under any of the stresses applied as compared to the concentration obtained under control conditions, with the maximum (two-fold) found when salinity and heat were applied jointly. RIL-66 only had an increase in amino acids concentration under the combination of salinity and heat (**Figure 4F**). Due to these results obtained, a complete accumulation profile of the most physiologically-important amino acids in the tolerant and sensitive tomato lines under the different stresses studied was performed (**Figure 5**). The results are shown in **Figure 5A** and these were expressed as log_2_ value of the normalized concentration obtained under the different stresses applied against the control (**Supplementary Table S2**).

### Amino Acids Profile

As observed in **Figure 4F**, the different stresses resulted in a different amino acid accumulation profiles. Heat applied as a unique stress was the most similar to control plants and only gamma-aminobutyric acid (GABA), alanine (Ala), and glutamine (Glu) showed an inhibition of their accumulation in the sensitive line RIL-66, whereas proline (Pro) and asparagine (Asn) increased significantly in both tolerant and sensitive recombinant lines as compared to their controls (**Figure 5A**; **Supplementary Table S2**). On the other hand, salinity and the combination of salinity and heat induced changes in most of the amino acids studied. In general terms, salinity seems to significantly reduce the accumulation of most of the amino acids in both recombinant lines, except for Pro (in RIL-66 and RIL-76), and Tyr and Glu (in RIL-76) (**Figure 5A**). The combination of salinity and heat induced a specific response in the accumulation of amino acids depending on the recombinant line studied. Thus, Pro was significantly accumulated in both lines, as described under salinity stress. However, many amino acids showed an antagonistic regulation in the sensitive line RIL-66 as compared to the tolerant RIL-76 under salinity+heat combined. Thus, the accumulation of GABA, Asn, and Gln was lower in RIL-66 but higher in RIL-76, whereas arginine (Arg) was highly accumulated in RIL-66 but not in RIL-76 in this treatment as compared to their controls (**Figure 5A**). Due to the involvement of Arg, Glu, Gln, and Asn in the N metabolism in plants and the results obtained and described above for these amino acids (**Figure 5A**), a detailed and individual study was performed for these four (**Figures 5B–E**).

Arg showed significant lower concentrations in plants grown under salinity as a sole stress in both recombinant lines, although even lower in RIL-76. On the contrary, under salinity+heat, RIL-66 accumulated the highest amount of Arg (around 70% more that its control), whereas RIL-76 showed the lowest Arg concentration (56% less than its control) as compared to the rest of the treatments. Glu was significantly lower in the sensitive line RIL-66 under salinity, heat, or the combination of salinity+heat with respect to its control, whereas in the tolerant RIL-76, Glu reached its highest value under salinity (~40% more than control plants) followed by the salinity+heat treatment (~23% more than control). Heat stress applied as a single stress did not induce any changes in Glu concentration in RIL-76 as compared to control plants. Gln and Asn showed a similar profile in both recombinant tomato lines studied under the different stresses applied. Thus, salinity induced a significant reduction in the concentration of these two amino acids in both lines. RIL-66 showed an increase in the concentration of both amino acids under heat stress, whereas RIL-76 showed this increase only for the amino acid Asn, with Gln being significantly reduced with respect to control plants. Interestingly, the application of salinity and heat simultaneously gave different results depending on the tomato line studied. Thus, RIL-66 showed a reduction in the foliar concentration of these amino acids, whereas RIL-76 showed an antagonistic behavior, with the highest accumulation for Gln and Asn under these conditions, doubling the concentrations found under control conditions.

### Nitrogen Metabolism-Related Genes and Enzymes

**Figure 6:**
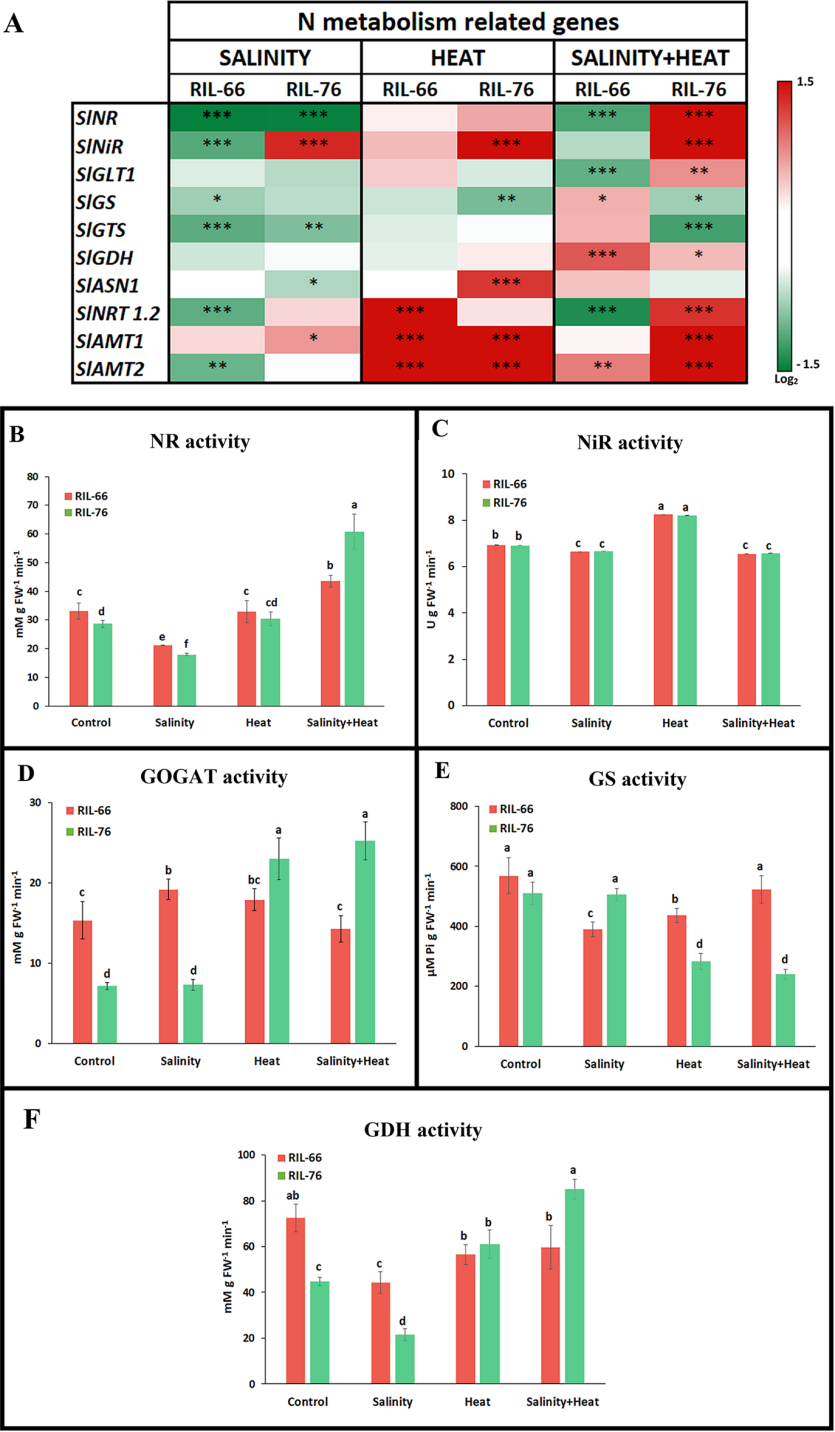
Expression and activities of the genes and enzymes involved in nitrogen (N) assimilation pathway. **(A)** Heat map of the expression of the N-metabolism related transcripts in leaves of RIL-76 (salt + heat tolerant) and RIL-66 (salt+heat sensitive) under salinity, heat, or the combination of salinity and heat. Scale is log_2_ of the mean values after normalization against control plants (n = 6). Asterisks are representative of significant differences at p < 0.05 (*p < 0.05; **p < 0.01; ***p < 0.001). **(B**–**F)**Absolute activities of the N-assimilation pathway related enzymes in recombinant inbred line (RIL)-66 and RIL-76 under control, salinity, heat, or the combination of salt+heat. Bars are mean values ± SE (n = 6), and bars with different letters are significantly different at p < 0.05 according to Tukey's test. (n = 6).

To contribute with new knowledge on the specificity of abiotic stress combination on nitrogen metabolism, the expression of the main nitrogen metabolism-related transcripts and the activity of the main enzymes involved in this pathway were measured under the different conditions used in our experiments (**Figure 6**). As expected, salinity and heat stress applied in combination resulted in the induction of significant and important changes in the expression of most transcripts under evaluation with respect to the control plants, and these changes could not be observed when in the expression of these genes when these stresses were applied alone (**Figure 6A**, **Supplementary Table S4**).

In general, salinity induced a down-regulation of most of the N-related transcripts studied in both recombinant lines, with two exceptions: in the tolerant line RIL-76, the genes encoding for nitrite reductase (*SlNiR*) and for ammonium transporter 1 (*SlAMT1*) were upregulated as compared to control plants, whereas in the sensitive line RIL-66 these transcripts were down-regulated by salinity (**Figure 6A**). Under heat, most of the transcripts that showed significant differences with respect to control plants were up-regulated, with the exception of the gene that coded for glutamine synthase (*SlGS*), which was down-regulated in RIL-76 as compared to control plants. Thus, the ammonium transporters *SlAMT1* and *SlAMT2* were significantly up-regulated in both recombinant lines, nitrate transporter 1.2 (*SlNRT1.2*) was up-regulated in the sensitive RIL-66 line and asparagine synthase 1 (*SlASN1)* and nitrite reductase (*SlNiR*) were up-regulated in the tolerant RIL-76. As indicated previously, when salinity and heat were applied to these two recombinant tomato lines jointly, the response obtained for the N-related transcripts studied was different to that observed when these stresses were applied individually and it was not deducible from the individual responses obtained. In this case, most of the transcripts showed an antagonistic response depending on the recombinant line studied. Thus, *SlNR, SlNiR, SlGLT1*, and *SlNRT1.2* were down-regulated in the sensitive RIL-66 whereas in the tolerant RIL-76 those transcripts showed a significant up-regulation with respect to control plants. On the contrary, *SlGS* and *SlGTS* were up-regulated in RIL-66 but down-regulated in RIL-76. Only two transcripts showed a synergistic up-regulation under the combination of salinity and heat, *SlGDH* and *SlAMT2* (**Figure 6A**; **Supplementary Table S4**).

As of now, our results point to a differential regulation of the nitrogen metabolism between the sensitive and the tolerant recombinant lines under the stress conditions studied here, with significant differences in the transcriptional regulation of the N-related genes. To better understand if this differential regulation was not only at the transcriptional level, but also at post-transcriptional level, the activities of the main enzymes involved in the N assimilation pathway were studied. Our results showed that NR, NiR, GS, GOGAT, and GDH activities were differentially modified by temperature and salinity stresses as well as for their combination. NR activity reached its highest value in the tolerant line RIL-76 under the combination of salinity and heat (**Figure 6B**), being 2.5-fold higher that the activity found in this recombinant line under control conditions. The sensitive RIL-66 also showed a NR activity that was higher under the treatment salt+heat than under the other conditions used in our experiments, but this activity was just 26% higher than its respective control. Curiously, salinity applied as a single stress induced a, significant inhibition in NR activity in both recombinant lines as compared to their control plants (**Figure 6B**).

NiR activity was similar in both recombinant lines under the different conditions used (**Figure 6C**). Thus, under heat, NiR activity significantly increased by 16%, whereas salinity and the combination of salt+heat induced a small but significant reduction of the NiR activity of 5% with respect to the control plants (**Figure 6C**).

RIL-66 showed a higher GOGAT activity than the one found in RIL-76 under control conditions, being 53% lower in the tolerant RIL-76 (**Figure 6D**). When the plants were subjected to salinity or heat as singles stresses, GOGAT activity increased by 20% in RIL-66 with respect to control plants. Under salinity stress, RIL-76 did not change significantly with respect to their control plants, but under heat or under the combination of salt+heat treatments, GOGAT activity increased 3.5 fold with respect to its control. With this last treatment (salinity+heat) RIL-76 had a GOGAT activity that was 1.75-fold higher than the one found in RIL-66 (**Figure 6D**). As for GS activity, both lines showed a similar activity under control conditions (**Figure 6E**). However, RIL-66 and RIL-76 differed depending on the stress applied. Thus, under salinity and heat applied as individual stresses, RIL-66 showed a reduction in GS activity of 39 and 24%, respectively, as compared to that obtained under control conditions. The application of salinity and heat in combination did not induced any changes of this activity in RIL-66. On the contrary, in the tolerant RIL-76, GS activity did not change when salinity was applied as a sole stress, but it was significantly reduced by about 45% under heat or the combination of salinity+heat (**Figure 6E**). Finally, GDH activity showed very different profiles depending on the recombinant line studied (**Figure 6F**). Thus, RIL-76 resulted in a GDH activity that was 38% lower than the one found for RIL-66 under control conditions. Under salinity, RIL-66 and RIL-76 showed an inhibition of GSH activity of 38 and 52%, respectively, as compared to their control plants. However, under heat or the combination of salt+heat RIL-66 showed a GDH activity that was about 18% lower than its control, whereas RIL-76 showed a 1.3-fold increase of this activity under heat and a 1.9-fold increase under the combination of salinity and heat (**Figure 6F**).

## Discussion

**Figure 7:**
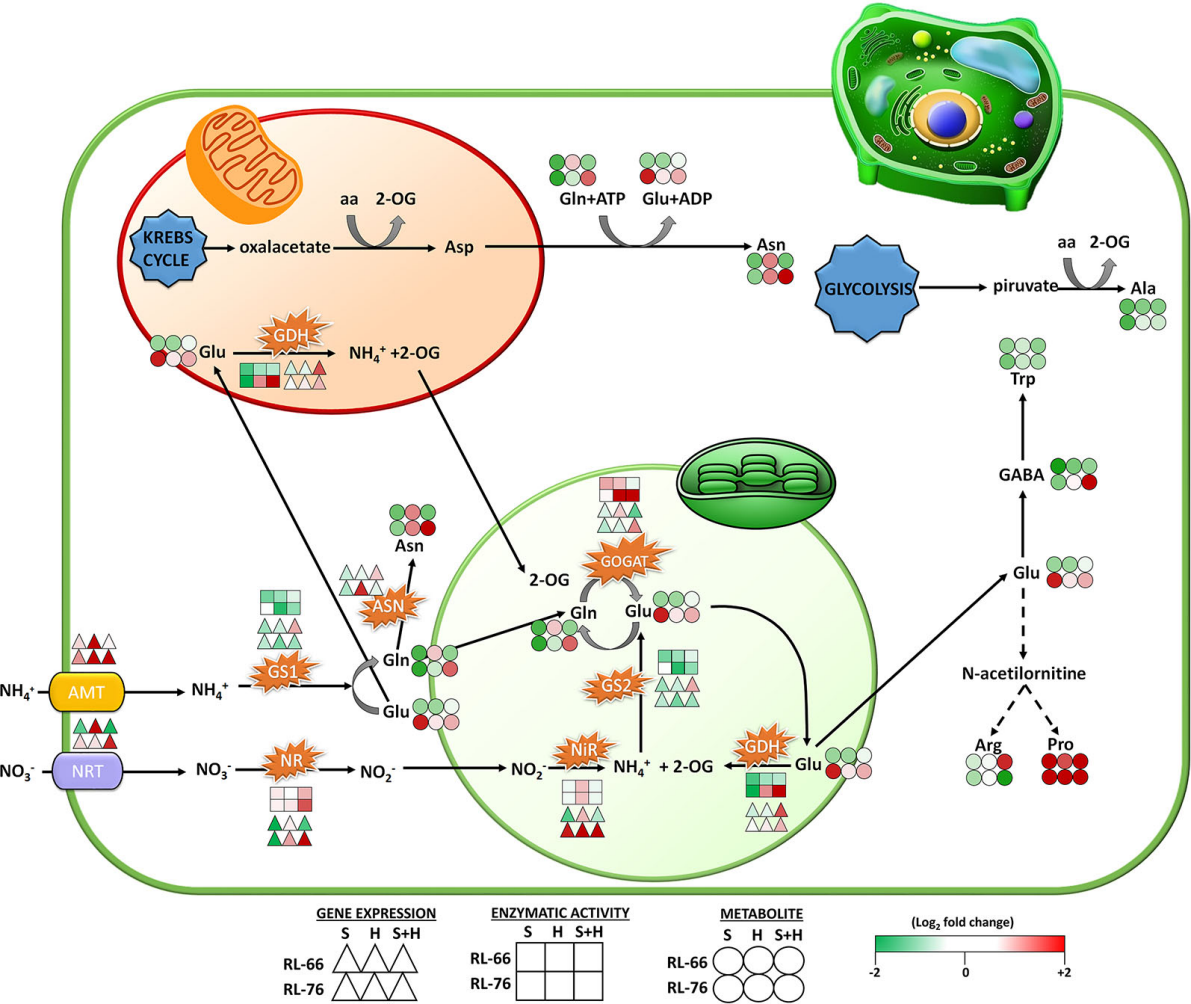
Scheme of the proposed changes occurring in the nitrogen (N)-assimilation pathway. Changes were represented in recombinant inbred line (RIL)-66 (salt+heat sensitive; up row symbols) and RIL-76 (salt+heat tolerant; down row symbols) under salinity, heat, or the combination of salinity and heat at gene (Δ), enzyme (□), and metabolite (○) levels. Changes are expressed as the log2 of the normalized values against the respective control values obtained in each recombinant line (n = 6).

Nitrogen (N), as a primary plant nutrient, determines the growth of the plant and its productivity. It is required by plants for the synthesis of key compounds such as amino acids, nucleic acids, proteins, and chlorophyll. Most plant species are capable of utilizing N in the forms of nitrate (NO_3_^−^) and ammonium (NH_4_^+^). In plants, the active uptake of NO_3_^−^ and NH_4_^+^ is mediated by NRTs and AMTs, respectively (Debouba et al., 2007; Goel and Singh, 2015). After its uptake, nitrogen assimilation continues with the reduction of NO_3_^−^ to NH_4_^+^, with the involvement of several enzymes (such as NR, NiR, GS, among others) (**Figure 7**), with NH_4_^+^ finally incorporated into amino acids through the process of ammonia assimilation (Ferrario-Méry et al., 1998; Hoshida et al., 2000; Debouba et al., 2007). In plants, several processes such as N uptake and assimilation have been shown to be severely affected by abiotic stresses such as salinity, drought, and extreme temperatures. There are many experimental works in the literature that show how a single abiotic stress can affect N uptake and assimilation at the physiological, biochemical, but also at gene expression level (Hemon et al., 1990; Mungur et al., 2005; Ashraf et al., 2018). However, very little information can be found about how the combination of two or more abiotic stresses may affect this important metabolic process (Cui et al., 2019). In recent years, an increase in the experiments in the field of abiotic stress combination has been observed, most probably due to the current undeniable effects of climate change on our agriculture and to the fact that in nature, abiotic stresses always act in combination. However, and with N metabolism being one of the most important pathways in plants due to its direct involvement in plant growth, development, and production, it is surprising the lack of information about how abiotic stresses acting in combination might affect this pathway. Therefore, the aim of this work was to increase the knowledge on how the combination of salinity and heat, two of the most devastating abiotic stresses acting jointly in a vast area in the world's agricultural lands, affected N metabolism and its related parameters. The use of a tomato RIL population composed of individuals with different degrees of tolerance to combined abiotic stresses together with the dissection of this specific pathway, may help to identify which elements (genes, metabolites, or enzymes) are more susceptible to these environmental conditions, which are directly related to climate change.

### Growth and Photosynthesis Were Differently Affected in the Sensitive and the Tolerant Recombinant Lines Under the Combination of Salinity and Heat

In our experiments, from a RIL population of 84 lines differing in their tolerance to the combination of salinity and heat, 2 contrasting lines (sensitive, RIL-66; high tolerant, RIL-76) were selected to perform our experiments under combined, but also under single stresses, with the aim of better understanding if the combination of salinity and heat had a specific response pattern that was somewhat different from the observed under simple stress. As expected, the sensitive line RIL-66 showed a significant decrease in biomass production under salinity and salinity+heat as compared to plants grown under control or under heat, or to the tolerant RIL-76, which confirmed that RIL-66 was a sensitive line, not only to the combination of salinity and heat (as selected for) but also to salinity. Along the same line, RIL-76 was confirmed as a very tolerant line to simple or combined stresses.

Under salinity, the decrease in biomass observed in RIL-66 was correlated with an inhibition of all the photosynthetic and chlorophyll fluorescence parameters, in agreement with previous publications (Yu et al., 2010; Ashraf et al., 2018; Naliwajski and Skłodowska, 2018). However, the inhibition induced by the application of salinity+heat combined in this RIL-66 was observed only in the CO_2_ assimilation index and in the maximum quantum yield efficiency rate (Fv´/Fm´), with the rest of the photosynthetic parameters measured being very similar to those obtained under control conditions. These results might indicate that the inhibition of photosynthesis in RIL-66 was independent of stomatal limitations, and it could be due to a downstream inhibition of Rubisco or triose phosphates utilization (Rivero et al., 2010). The fact that RIL-76 showed an increase in stomatal conductance, transpiration and electron transport rate (ETR) under stress combination may support the hypothesis that tomato sensitivity to salinity or salinity+heat combined observed in RIL-66 could be linked to photosynthesis-related enzymatic inhibition (Rivero et al., 2010; Zhou et al., 2011; Wang et al., 2018).

### The Combination of Salinity and Heat Induced a Deficiency on K^+^, Mg^2+^, and Organic N Content in the Sensitive Tomato Line

It has been previously shown that abiotic stress, such as salinity and/or heat, also induced changes in nutrients uptake. The increase in Na^+^ uptake induced under salinity stress generates an antagonistic effect on the absorption of other ions, with K^+^, Ca^+2^, Mg^+2^, and NO_3_^−^ being particularly affected under these conditions (Yildirim et al., 2009). The sensitive RIL-66 showed a significant decrease of K^+^ and Mg^2+^ under salinity and salinity +heat, whereas RIL-76 only showed a slight decrease on K^+^ concentration under salinity stress and Ca^2+^ under the combination of salinity and heat (Wang et al., 2013; Gouiaa and Khoudi, 2015; Plaza et al., 2017).

Na^+^ acts as an antagonistic ion of NO_3_^−^. Our results showed that the application of most of our treatments significantly reduced the NO_3_^−^ concentration in both recombinant lines, except for RIL-66 grown under salinity, where this concentration was not significantly different from control plants. The accumulation of NO_3_^−^ in those edible plant parts (such as fruits in tomato or leaves in lettuce, for instance) has been defined as not healthy for human consumption due to its relationship with the development of cancer and tumors (Dutt et al., 1987; Du et al., 2007). Since leaves are the main source of nutrients for tomato fruit loading, the significant decrease of this N form observed in all the stress treatments applied with respect to control plants could indicate a positive effect of these stresses on NO_3_^−^ assimilation into proteins and amino acids. However, these observed decreases could be also due to a reduction in N absorption from the roots induced by these stress conditions. Thus, total N and organic N content in these leaves where also measured. Our results indicated that only the application of salinity (for total and organic N) and salt+heat (for organic N) on RIL-66 negatively affected N uptake and accumulation as total or organic N, with organic N defined as N contained in proteins and amino acids.

### Nitrogen Metabolism was Improved Under the Combination of Salinity and Heat in the Tolerant Line at Transcriptional and Protein-Related Levels

The fact that total amino acids concentration increased under any stress condition used in both sensitive and tolerant recombinant lines, with a significant increase observed in the tolerant RIL-76 under the combination of salinity and heat, made us delve into and investigate the concentration of the most metabolically-important amino acids under these conditions. The profile obtained under simple or combined stresses for both tomato recombinant lines is clearly indicative that the application of abiotic stresses in combination induced a specific response in the accumulation of amino acids profile (**Figure 5A**). The only amino acid that was consistently accumulated under single or combined stresses and in both recombinant lines was Pro. Pro has been previously described as a N-rich amino acid that can act as a compatible solute (Rivero et al., 2004; Hayat et al., 2012). Compatible solutes are organic compounds with a high level of solubility and a low molecular weight that are usually non-toxic for the cells when they accumulated at high concentrations, and are usually found in cells suffering from environmental stresses. When accumulated under stress, they protect the cells by contributing to the osmotic adjustment, membrane protection, reactive oxygen species (ROS) detoxification, and protein stabilization (Bohnert and Jensen, 1996; Yancey, 2005; Ashraf et al., 2018). Therefore, the accumulation of Pro observed in our recombinant tomato lines may be explained by its osmoprotectant role within the cells under the environmental stress conditions used.

From the amino acids profile obtained and shown in **Figure 5A**, it was interesting to look into those that had an antagonistic accumulation pattern between the tolerant and the sensitive lines under the different scenarios used, in order to find a possible marker that could be linked to this abiotic stress combination tolerance. Interestingly, from the amino acids studied, GABA, Tyr, Asn, Arg, Glu, and Gln were those with antagonistic accumulation patterns. Thus, GABA, Val, Asn, and Gln significantly accumulated only in RIL-76 when those plants were grown under the combination of salinity and heat. On the other hand, Arg accumulated in the sensitive RIL-66 under stress combination and not under the other stresses applied, whereas in the tolerant RIL-76, the concentration found for this amino acid under salinity+heat was significantly lower than control plants. Glu was only accumulated under salinity in the tolerant RIL-76 and Val and Ile were also found at high concentrations in RIL-76 under the combination of salt+heat but not under the other conditions. This differential accumulation may have a biological sense, and curiously, most of the differentially-accumulated amino acids were directly related to the N assimilation pathway, such as Gln, Asn, Glu and Arg. Jin et al. (2016) found that in *P. oleracea* L. plants there was a specific accumulation of certain amino acids, such as Glu, Tyr, Val, and Trp when subjected to drought and heat stress acting in combination, which is also in accordance with the results found in our experiments. These authors suggested that the specific accumulation of these amino acids might play a role in the cellular osmotic adjustment aimed to keep the leaf turgor during this stress condition. Glu, Gln, and Asn are the most important amino acids resulting from the N assimilation in plants. NO_3_^−^ is transported into the cells by the NTRs and reduced to NH_4_^+^ by NR and NiR. Also, NH_4_^+^ can be transported directly into the cells through AMTs and thus NH_4_^+^ is incorporated into the N assimilation pathway (**Figure 7**). The N assimilation pathway results in the synthesis of certain important amino acids, such as Glu, Gln, Asn, and Asp that are necessary for the correct functioning, gearing, and interplay of others pathways that are part of the cellular metabolism.

This assimilation can take place in the cytoplasm or in different cell compartments, such as the plastids or the chloroplast (Krapp, 2015), where the resulting amino acids can be necessary for other functions or transported to other cell sites. NH_4_^+^, together with C skeletons, is used by the plant to produce various amino acids with the GS/GOGAT cycle. The activity of N-assimilatory enzymes such as nitrate and ammonium transporters can be regulated at a number of different biochemical steps, i.e., during the synthesis of messenger RNA (mRNA) and protein (transcription and translation, respectively) and through the modulation of the enzyme's activity (post-translation). Also, the transcription and/or translation/post-translation regulation of these proteins can be seriously affected by abiotic stresses. Previous work has shown that the negative effect of NaCl on the N assimilation of plants is strictly related to modifications of the enzymes involved in the NO_3_^−^ assimilation pathway induced by salinity (Debouba et al., 2006; Debouba et al., 2007; Ehlting et al., 2007; Flores et al., 2011). In roots and leaves of beans, as significant decrease of NR activity was observed (Gouia et al., 1994), as well as on leaves of maize (Baki et al., 2000), sugar beet (Ghoulam et al., 2002), and tomato (Debouba et al., 2007) after the plants were exposed high concentrations of NaCl in the environment for 5 to 10 days. On the contrary, a study also demonstrated the stimulatory effect of salinity stress on the NR activity in sprouting bean seeds (Misra and Dwivedi, 1990), soybean roots (Bourgeais-Chaillou et al., 1992), and tomato roots (Debouba et al., 2007). Conversely, the activity of the enzyme was unaffected in poplar plants treated with NaCl for 1 or 2 weeks. Our experiments revealed an inhibition of NR activity (**Figure 6B**) under salinity stress in both recombinant lines, which was induced at the transcriptomic level (**Figure 6A**). A decrease in NR activity observed during long-term (more than 24 h) salinity stress has been suggested to be the result of reduced NO_3_^−^ uptake (Flores et al., 2011), lower NO_3_^−^ content in tissues, and a decreased expression of NR genes (Gouia et al., 1994). Indeed, a high concentration of NaCl in the environment led to the significant decrease of NO_3_^−^ uptake in wheat (Botella et al., 1997), tobacco (M. Ruiz et al., 2005), bean (Gouia et al., 1994), and tomato (Flores et al., 2011). Our results showed that NO_3_^−^ concentration in leaves was reduced in RIL-76 under any of the stresses applied, but in RIL-66 it was found lower only under heat applied as an individual stress. Modifications of NR activity under salinity have been linked with an increase in the enzyme's activation status in roots and a decrease in the pool of phosphorylated enzyme in the cytosol (Di Gioia et al., 2017). The decrease in ATP levels in cucumber root cells due to salinity stress could also limit the activity of the protein kinase. Therefore, the temporary decrease of cytosolic pH during salinity stress may result in the increase in NR activity in roots *via* the dephosphorylation of the NR protein. In their study with spinach, Huber et al. (1994) described a strong increase in NR activity in leaves after the addition of different salts (both organic and inorganic) into the extracts which contained the enzymes. The authors concluded that the stimulation of NR could have been due to the presence of salts and the effect of the high ionic strength created.

There are other works that showed how high salinity inhibited the activity of many enzymes involved in N assimilation in maize, mung bean, and tomato (Khan and Srivastava, 1998; Chakrabarti and Mukherji, 2003; Debouba et al., 2006; Debouba et al., 2007). Also, increased levels of salinity were shown to reduce the activities of various nitrogen assimilation-related enzymes such as NR, NiR, GS, GOGAT, and GDH in *Brassica juncea* (Nathawat et al., 2005; Siddiqui et al., 2009). Likewise, the activities of NR and GS in barley under drought stress were found to be reduced (Robredo et al., 2011). As for wheat, the translocation of N during the grain filling period was shown to be limited under drought stress, resulting in lower grain yields (Kirda et al., 2001). Also, in creeping bentgrass, wheat, and rice high temperature was shown to inhibit the uptake and assimilation of NO_3_^−^ (Ercoli et al., 2008; Ito et al., 2009). In another study which utilized *NR-*gene overexpressing transgenic tobacco under drought stress, the plants retained 50% of NR activity as compared to untransformed plants, with NR activity not detected in untransformed plants (Ferrario-Méry et al., 1998). Lastly, in a study where chloroplast *GS2* was overexpressed, enhanced salt tolerance in transgenic rice was found as a result (Hoshida et al., 2000), while the ectopic overexpression of pine cytoplasmic *GS1* in transgenic poplar conferred these plants with an enhanced tolerance to drought stress (el-Khatib et al., 2004). On the contrary in rice, the overexpression of the *OsGS1;2* gene resulted in a higher sensitivity to salt, drought and cold stress (Cai et al., 2009). Also in rice, the overexpression of the *OsGS* gene enhanced tolerance to cadmium stress due to oxidative stress response modulation (Lee et al., 2013). Overexpression of *Escherichia coli gdhA* conferred tolerance under water deficit conditions in transgenic tobacco and maize (Mungur et al., 2005). Most of these studies also correlated an improvement of growth and plant production when N assimilation increased. Our results showed that GOGAT, GS, and GDH showed different behaviors depending if the tomato line was sensitive or tolerant to the combination of salinity and heat. Thus, GOGAT was inhibited in RIL-66 and overexpressed in RIL-76; GS (cytoplasmic, *SlGTS1)* was strongly inhibited in RIL-76 under stress combination, whereas GDH was upregulated in both recombinant lines under these conditions. Our results of the gene expression, translated into enzymatic activity of the transcribed protein, revealed that GOGAT and GS were regulated at the transcriptional level, whereas GDH might have suffered a post-translational modification, as the activity found was much lower that the expression of their respective transcripts. Also, it is possible that under the combination of salinity and heat, the low accessibility of glutamate by GS may be due to glutamate being diverted to the synthesis of osmolytes under such conditions, such as Pro (**Figure 7**) (Ashraf et al., 2018). The clear reduction of the NO_3_^−^ absorption and the osmotic changes induced by salinity but also under salt+heat combination in RIL-66 may also be responsible for the inhibition of the activities of most of the N-assimilation related enzymes under these conditions, as Ashraf et al. (2018) reported previously.

## Conclusion

Traditionally, the selection of the different agronomical plant varieties has been done accordingly to their vigor and yield. However, the new climate scenario is forcing us to select plant varieties based on their tolerance to new environmental conditions. In our experiments, the improvement in NR, NiR, GS, and ASN enzymatic activities found in the tolerant line RIL-76 clearly correlated with the high levels of Gln, Glu, and Asn, as well as with a better performance of the photosynthetic apparatus and with a higher growth under the combination of salinity and heat [see principal component analysis (PCA) and dendrograms in **Supplementary Figures S1–S3**]. Our results demonstrate that these can be used as molecular markers for the selection of varieties with improved NUE and thus, increasing plant productivity under abiotic stress combination. These results can help scientific community to alleviate the negative effects of climate change on plant yield and can also serve for reducing the abuse of N in plant fertilizers by improving N use efficiency under these unfavorable conditions.

## Data Availability Statement

All datasets generated for this study are included in the article/**Supplementary Material**.

## Author Contributions

ML-D and RMR conceived the idea and wrote the manuscript. ML-D also participated and supervised the physiological, ionomics and biochemistry experiments. DC and MG-M carried out the biochemistry experiments. PN, MN-C, VM, and RMR carried out the physiological experiments. FR and RMR carried out the ionomics experiments, while ML-D, RM, and RMR made the gene expression analyses.

## Funding

This work was supported by the Ministry of Economy and Competitiveness from Spain (Grant No. AGL2015-66033-R and Grant No. PGC2018-095731-B-I00, MCIU/AEI/FEDER, UE).

## Conflict of Interest

The authors declare that the research was conducted in the absence of any commercial or financial relationships that could be construed as a potential conflict of interest.
