# Supplementary material for: Using Tomato Recombinant Lines to Improve Plant Tolerance to Stress Combination Through a More Efficient Nitrogen Metabolism
Source: Front Plant Sci. 2020 Jan 17;10:1702. doi: 10.3389/fpls.2019.01702 (PMC6983915; doi:10.3389/fpls.2019.01702)

Supplemental Scheme 1. Experimental design for the three experiments carried out for the selection and experimentation with the tomato RILs population

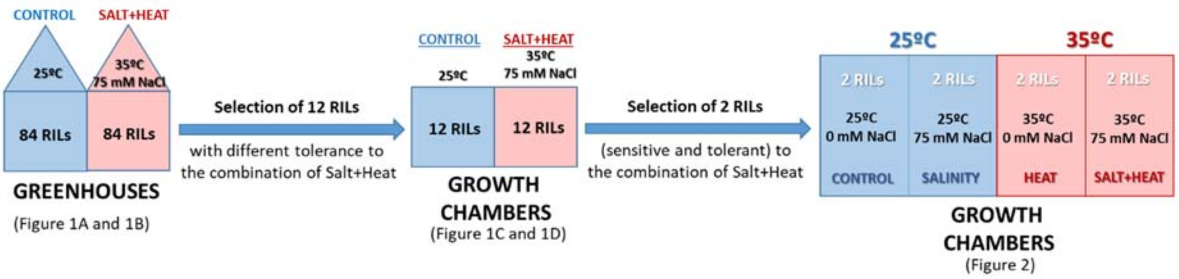

Supplemental Table S1. Absolute values obtained in cations concentration (µmol/g DW) in leaves of two tomato recombinant lines (RIL-66 and RIL-76) under control, simple stress (salinity or heat) or the combination of salinity+heat. Values are means (n=6)

Treatment	Recomb. Line	Na^+	K^+	Ca^{2+}	Mg^{2+}
Control	RIL-66	2.47234158	244.607857	113.450981	28.9775737
	RIL-76	2.98536357	308.253496	127.330764	28.4618876
Salinity	RIL-66	110.821929	104.709183	110.388532	11.3756017
	RIL-76	146.06509	158.638543	124.314263	26.8133111
Heat	RIL-66	3.22416865	319.116882	115.430588	28.9804243
	RIL-76	3.10699108	295.30514	117.081159	29.4970716
Sal+Heat	RIL-66	82.6482231	106.63768	90.7005475	12.7424652
	RIL-76	101.407195	291.575017	86.5951295	26.6791909

Supplementary Table S2. Absolute values obtained in amino acids concentration (µmol/g DW) in leaves of two tomato recombinant lines (RIL-66 and RIL-76) under control, simple stress (salinity or heat) or the combination of salinity+heat. Values are means (n=6)

		µmol/g DW												
Treatment	Recomb. Line	GABA	Ala	Val	Pro	Asn	Trp	Tyr	Isoleu	Thr	Arg	Glu	Phe	Gln
CONTROL	RIL-66	0.39	1.26	0.08	0.49	1.99	0.03	0.03	0.11	0.38	8.69	27.49	0.08	7.12
	RIL-76	0.41	1.14	0.07	0.56	1.69	0.03	0.03	0.08	0.43	8.38	27.33	0.07	7.97
SALINITY	RIL-66	0.09	0.62	0.05	3.30	1.05	0.02	0.03	0.09	0.19	7.11	18.15	0.03	3.58
	RIL-76	0.23	0.51	0.05	2.79	1.03	0.01	0.13	0.08	0.18	5.76	40.62	0.02	3.14
HEAT	RIL-66	0.22	0.74	0.10	1.02	3.28	0.02	0.05	0.14	0.28	8.31	17.27	0.06	10.10
	RIL-76	0.43	0.99	0.09	2.36	2.79	0.02	0.04	0.09	0.34	8.18	28.77	0.05	6.38
SAL+HEAT	RIL-66	0.25	0.74	0.08	2.67	1.27	0.02	0.04	0.13	0.24	13.91	23.24	0.04	4.95
	RIL-76	1.54	0.91	0.14	3.48	4.61	0.02	0.05	0.14	0.24	3.45	33.45	0.04	14.93

Supplementary Table S3. Primers sequences of the N-metabolism related transcripts tested by qPCR in leaves of two tomato recombinant lines (RIL-66 and RIL-76). *SlEF1* was used as internal control for data normalization.

Gene		Accession	Sense primer sequence	Anitsense primer sequence
SlNR	Nitrate reductase	Solyc11g013810.1.1	GGTTCATCACTCCCGTACCACTT	TCTGCTTCACCATATTCTGCTCT
SlNii	Nitrite reductase	Solyc01g108630.2.1	GGATTCATGGGATGCCTGACTAGA	TTCTCGTGGAACTGCACCAAAGT
SlGLT1	Glutamate synthase, NADH/NADPH, small subunit 1	Solyc03g083440.2.1	GGGAGAGTTTGAGGGTAGGGAATA	CAAGCAAACTGGCACGCACATA
SlGS	Chloroplast Glutamine synthetase	Solyc01g080280.2.1	AGCTTGTACCTTGTTGAGTTTCCCT	CACCTCAACTCCAAAGTAGTCTCATCTCT
SlGTS1	Cytosolic Glutamine synthetase	Solyc10g083830.1.1	ACACGGAGAAGGAAGGAAAGGGAT	TCCGCGATCATAGAGGTCACAACA
SlGDH1	Glutamate dehydrogenase	Solyc10g078550.1.1	GTTTACTGGCTTGGACCTCAGTTT	CACTTTGTCCATGGGCAGAACTTACA
SlASN1	Asparagine synthase	Solyc04g055200.2.1	AACCTTGATCCTTCTGGTAGGGCT	TCGAGTCATGGACAGCAAGAGACT
SlNRT2.1	Nitrate transporter protein 2.1	Solyc11g069740.1.1	TCCAGTCAAGGGAACGGAAGAACA	ACCACGCTCTGATCGGCAATTT
SlAMT1.1	Ammonium transporter protein 1	Solyc09g090730.1.1	ATGTGGGTTCGTAGCGGCTTTAGT	TGCTGCTTCAAGTGGATCGTCGTA
SlAMT2	Ammonium transporter protein 2	Solyc04g050440.2.1	TTGTACCGCCGCTCTGACAACTTT	CATGGCTCAACAACTGCACAACCT
SlEIF1 α	Elongation factor 1α	Solyc06g005060.2.1	GAACTGTCCCAGTTGGTCGT	GTCAAACCAGTAGGGCCAAA

Supplementary Table S4. Log_2 of the expression of a selected set of transcripts related to the N-metabolism. Transcript expression was determined by qPCR (Applied Biosystems, Lincoln, NE) using the primers pair listed in Supplemental Table S3. Normalization of the expression was done using the internal and constitutive control *SlERF1* and samples normalization for log_2 calculation was performed with respect to control plants before stress (n=6).

	**SALINITY**		**HEAT**		**SALINITY+HEAT**	
	RIL-66	RIL-76	RIL-66	RIL-76	RIL-66	RIL-76
SlNR	-2.02624257	-1.48715496	0.1003437	0.74896037	-1.0749925	2.04905732
SlNiR	-1.01979764	1.34570217	0.42772611	2.1402998	-0.44874573	1.61988386
SlGLT1	-0.21411133	-0.43099817	0.31886355	-0.23856195	-0.93396346	0.89916852
SlGS	-0.67519976	-0.39715735	-0.31491915	-0.802543	0.49639257	-0.57151222
SlGTS	-0.97664611	-0.76421642	-0.19662507	-0.01933543	0.46724923	-1.12042618
SlGDH	-0.2961262	-0.04336866	-0.16730321	0.1210289	1.04047061	0.62991034
SlASN1	-0.3254877	-0.47721831	-0.0112547	1.25095113	0.38	-0.18233999
SlNRT 1.2	-0.9604084	0.26059062	3.53222046	0.18383471	-1.34827728	1.27987989
SlATM1	0.24454447	0.64554297	5.78692576	1.9982645	0.06776047	2.99190254
SlATM2	-0.89261818	-0.00200144	6.37705326	2.14450264	0.81360817	2.33149052

Supplementary Figure S1. Principal Component Analysis (PCA) in RIL-66. A PCA analysis was done with the 45 variables studied in this manuscript, with the components PC1, PC2 and PC· explaining 100% of the variability (PC1 and PC2 explained 80.10% of the variability). **PC1** (51.81%) explained FW, Pnet, gs, Na, K, Mg, Total-N, N-org, Total Soluble Proteins, Total Amino Acids Conc, Val, Asn, Trp, Glu, Phe, Gln, SlNR, *SlNiR, SlNTR1.2* and NiR activity. PC1 lets differentiate between the treatments without heat (salinity and control) against those treatments with heat as a factor (heat and salinity+heat). **PC2** (28.29%) explained Pnet, gs, Na, K, Mg, Total-N, N-org, Total Soluble Proteins, Total Amino Acids Conc, Val, Asn, Trp, Glu, Phe, Gln, *SlNR, SlNiR, SlNTR1.2* and NiR activity. PC2 lets differentiate between the treatments salinity+heat and control against heat and salinity. And **PC3** (19.90%) explained DW, ETR, N-NO3, GABA, Ala, Thr, Arg, SlGS, *SlAMT1, SlAMT2*, GOGAT activity, GS activity, GDH activity. PC3 lets differentiate between the treatments with salinity (salinity and salinity+heat) against those treatments without salinity as a factor (heat and control).

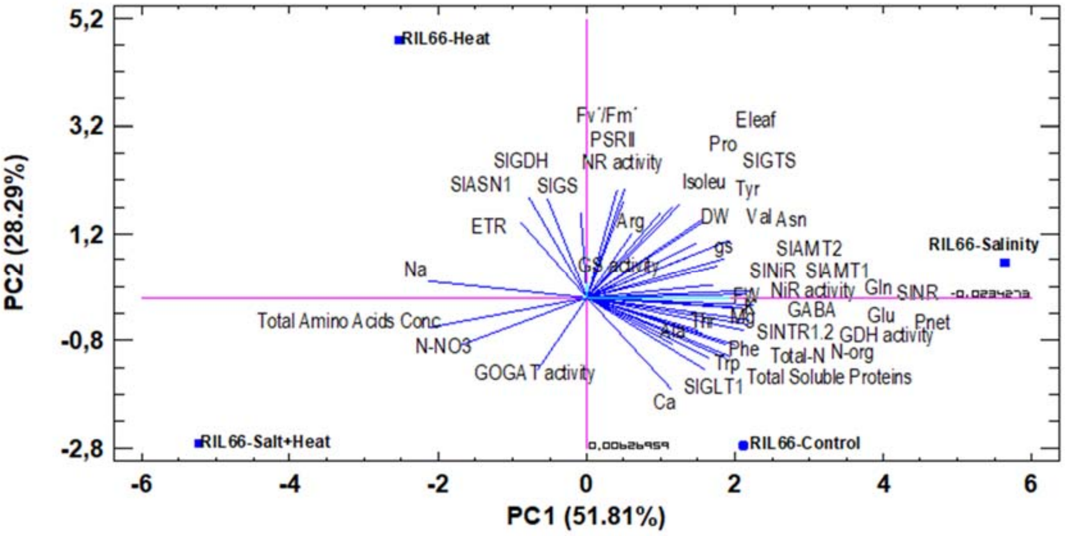

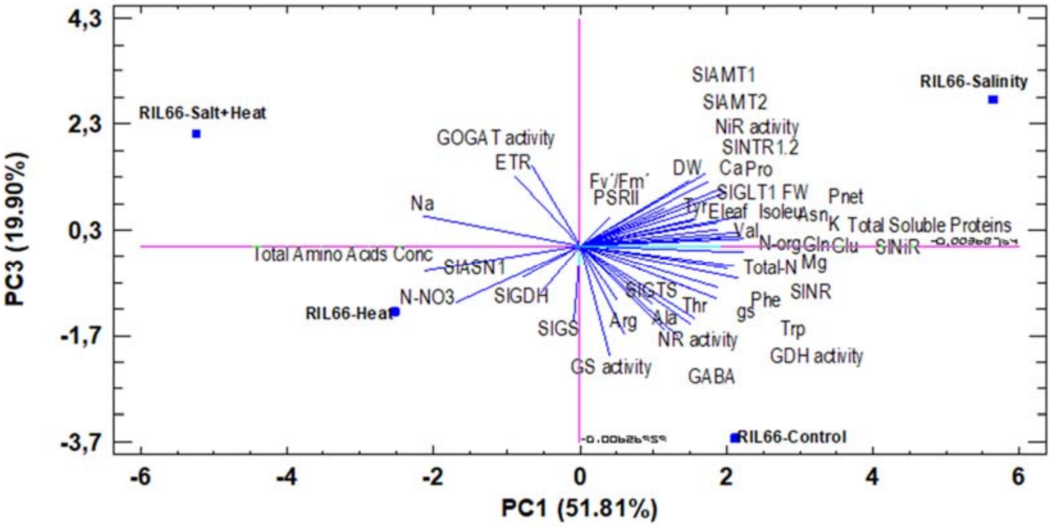

Supplementary Figure S2. Principal Component Analysis (PCA) in RIL-76. A PCA analysis was done with the 45 variables studied in this manuscript, with the components PC1, PC2 and PC· explaining 100% of the variability (PC1 and PC2 explained 78.93% of the variability). **PC1** (49.30%) Val, Pro, Asn, Isoleu, , gs, Eleaf, Ca, N-NO3, Arg, Glu, Gln, SlNR, SlAMT1, SlAMT2, SlNTR1.2, NR activity, GOGAT activity, GS activity, GDH activity, PSRII, ETR and GABA. This factor lets differentiate between the four treatments applied independiently. **PC2** (29.63%) explained Tyr, Thr, Phe, Fv´/Fm´, SlGTS, SlASN1, NiR activity, K, Mg, Na, Total-N, N-org, Total Soluble Proteins, Total Amino Acids Conc. PC2 lets differentiate between the treatments with heat (salinity+heat and heat) against those without heat as a factor (control and salinity). And **PC3** (21.07%) explained Ala, Trp, Gln, SlNiR, SlGS, DW, Pnet, Fv´/Fm´. PC3 lets differentiate between the treatments with salinity (salinity and salinity+heat) against those treatments without salinity as a factor (heat and control).

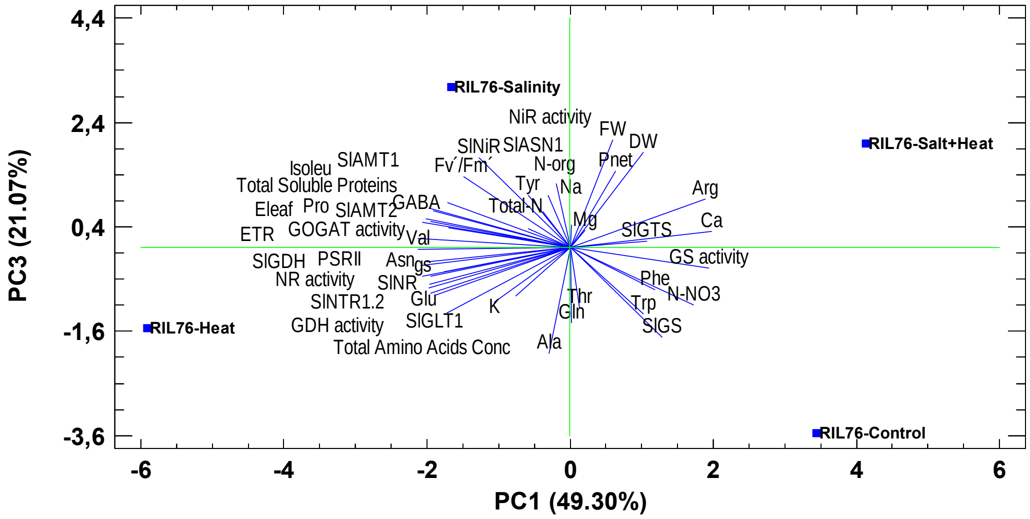

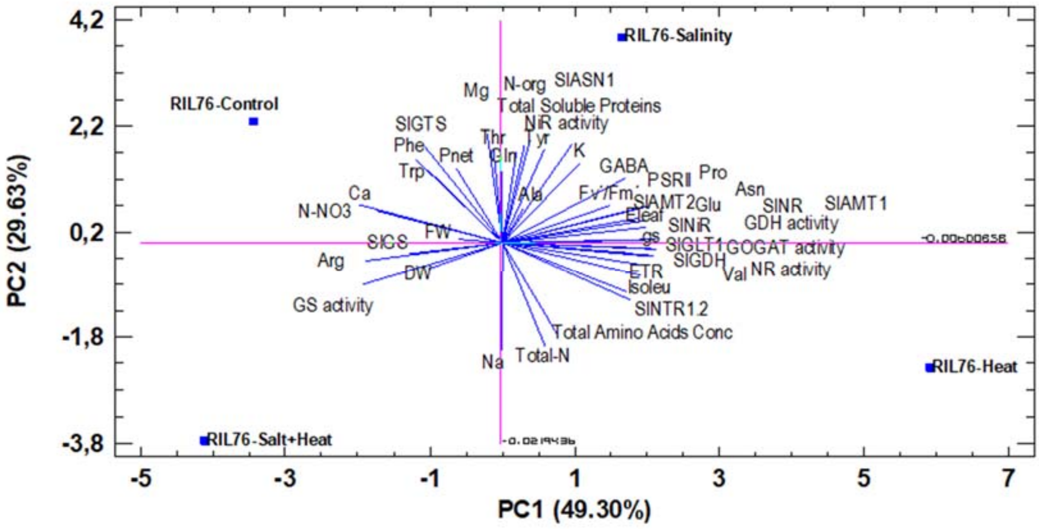

Supplementary Figure S3. **A.** Dendogram with Ward´s method for treatments separation in RIL-66. **B.** Dendogram with Ward´s method for treatments separation in RIL-/6.

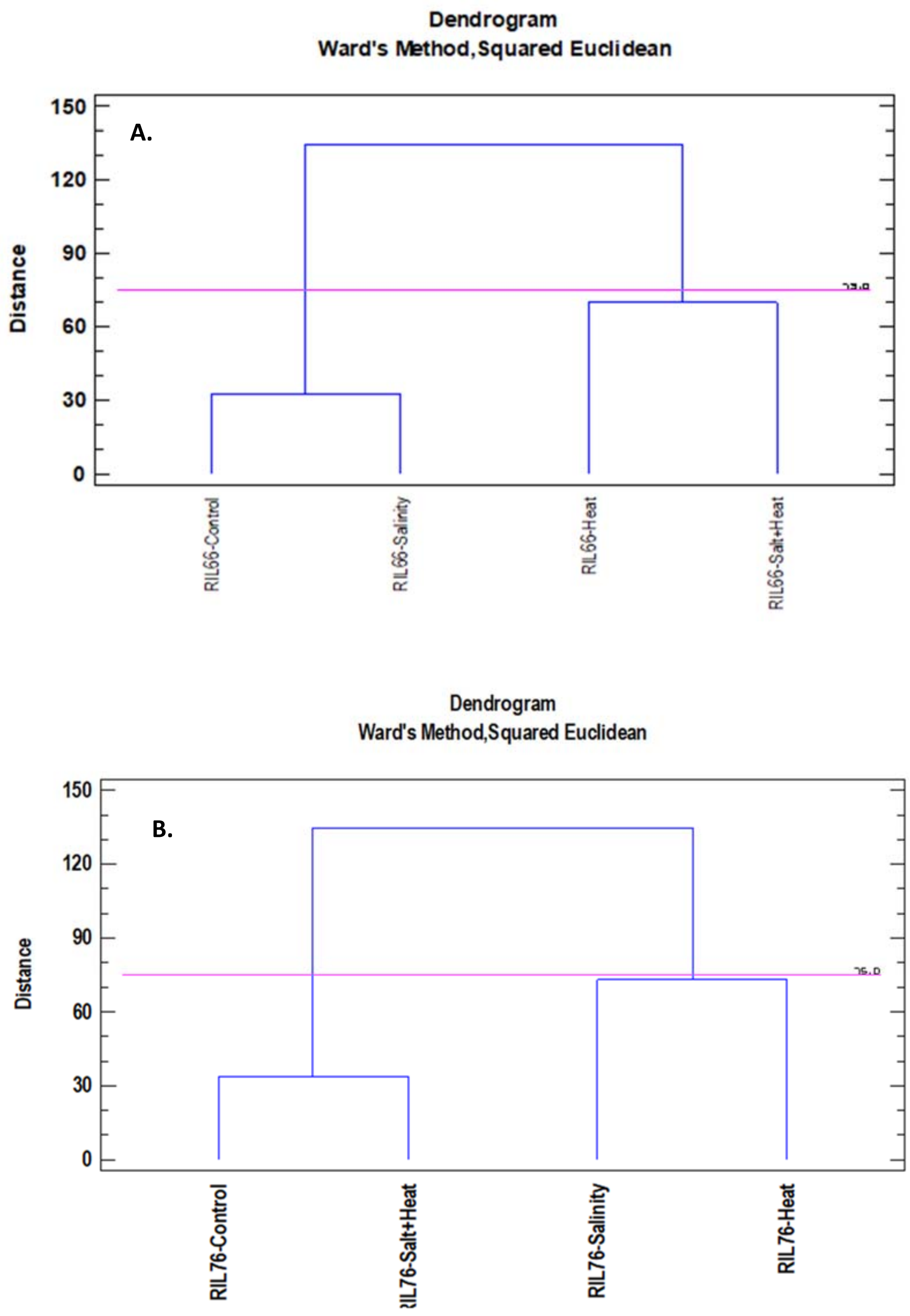

Supplement: Supplementary file 1 [file DataSheet_1.pdf]
